# Hierarchical Flowerlike 3D nanostructure of Co_3_O_4_@MnO_2_/N-doped Graphene oxide (NGO) hybrid composite for a high-performance supercapacitor

**DOI:** 10.1038/s41598-018-34905-7

**Published:** 2018-11-08

**Authors:** Sivalingam Ramesh, K. Karuppasamy, Hyun-Seok Kim, Heung Soo Kim, Joo-Hyung Kim

**Affiliations:** 1 Department of Mechanical, Robotics and Energy Engineering, Dongguk University–Seoul, Pildong-ro 1 gil, Jung-gu, Seoul, 04620 South Korea; 2 Division of Electronics and Electrical Engineering, Dongguk University–Seoul, Pildong-ro 1 gil, Jung-gu, Seoul, 04620 South Korea; 3 Department of Mechanical Engineering, Inha University, Inha-ro 100, Nam-gu, Incheon, 22212 South Korea

## Abstract

The present study investigates the fabrication of hierarchical 3D nanostructures with multi-component metal oxides in the presence of highly-porous graphene and characterized for its applications in high-performance supercapacitors. A hierarchical flowers like 3D nanostructure of Co_3_O_4_ @MnO_2_ on nitrogen-doped graphene oxide (NGO) hybrid composite was synthesized by thermal reduction process at 650 °C in the presence of ammonia and urea. The synthesized Co_3_O_4_@MnO_2_/NGO hybrid composites were studied *via* Raman, XRD, X-ray XPS, FE-SEM, FE-SEM with EDX, FE-TEM and BET analyses. The electrochemical analysis of Co_3_O_4_@MnO_2_/NGO hybrid composite electrode was investigated using cyclic voltammetry, chronopotentiometry and electrochemical impedance measurements. The hybrid composite electrode showed significant specific capacitance results of up to 347 F/g at 0.5 A/g and a corresponding energy density of 34.83 Wh kg^−1^ with better rate performance and excellent long-term cycling stability were achieved for 10,000 cycles. The obtained electrochemical results paved a way to utilize Co_3_O_4_@MnO_2_/NGO composite electrode as a promising electrode material in high performance supercapacitors.

## Introduction

Supercapacitors have received a great deal of consideration since they can comprise renewable energy storage devices with improved power density, cycling stability, low cost, and wide operating temperature ranges. In general, the carbon materials, metal oxides, metal hydroxides and conducting polymers are extensively employed as active materials for energy storage applications. Based on the electrochemical behaviors, the metal oxides have a high specific capacitance due to redox behaviors in the electrode materials. But, unfortunately, some of the metal hydroxides investigated earlier possess poor electrical conductivity that hinders the capacitive behavior^1–4^. In order to overcome the low capacitance behaviors, researchers have aimed to improve the electrical conductivity of the metal oxide with carbon materials for high-performance supercapacitors^5–7^. These hybrid composites have synergetic properties due to a combination of metal oxide or hydroxides in the presence of a redox reaction.

Recently, derivatives of graphene which include graphene oxide (GO), reduced graphene oxide (rGO) and N-doped GO have been utilized extensively for supercapacitor applications. These oxide functional moieties of graphene oxide interact with metal oxides to form M-O-M bonds which in turn facilitates the ionic mobility and diffusion rate and improves the performance of electrochemical double-layer capacitance (EDLC)^5–7^. Furthermore, the graphene and graphene oxides are chemically modified to improve the electrochemical performances^8–10^. The efficient chemical route is to dope the graphene with heteroatoms, such as nitrogen (N) and sulfur can be enhanced the electron mobility and capacitance in presence of valence electrons. Therefore, the NGO has attracted a significant amount of attention to synthesize the various chemical processes such as CVD and hydrothermal reactions.

Zhu *et al*. reported on CVD-derived NGO sheets by organic molecules *via* carbon and nitrogen atoms doped with higher concentration and growth temperature^11^. In addition, the carbon-based materials with a porous structure, transition-metal oxides and conjugated polymers are important active electrode materials for supercapacitors^12–14^. Transition metal oxides such as nickel (Ni), cobalt (Co), and manganese (Mn) oxides offer superior physiochemical properties and comprise excellent electrochemical supercapacitors^15^. In particular, cobalt oxide (Co_3_O_4_) is considered to be an auspicious electrode material for supercapacitors because of its economically cheap, high redox activity, high surface area and easily tunable surface properties. The nanostructured Co_3_O_4_ is the most stable cobalt oxide with a spinel structure and important p-type semiconductor which is widely used in Li-ion batteries, heterogeneous catalysts, electrochemical capacitors, electrochromic devices, solid-state sensors, solar selective absorbers for high-performance supercapacitors^15–17^. The hierarchical 3D structure provides a high surface area and rapid electron transport from the electroactive materials to the current collector, which may be due to the active material being grown directly on the current collector and avoiding the binders that normally cause a decrease in the electrode conductivity^17–21^.

Various types of MnO_2,_ MnO_2_/graphene, MnO_2_/Zn_2_SnO_4,_ MnO_2_/Cu, and MnO_2_/conducting polymers were used for supercapacitors applications^22–31^. Furthermore, the binary and ternary nanostructures of the hybrid composites are promising materials including Co_3_O_4_@MnO_2_, NiO/Ni core−shell, Ni(OH)_2_−MnO_2_/GO that have been widely used as electrodes for supercapacitors^32–35^. Therefore, the electrochemical properties of the nanostructured materials show a strong effect on the morphology to increase the specific capacitance and electro-catalysis towards capacitors, oxygen evolution (ORR) and (OER) reactions^36,37^.

Herein we report a hierarchical flower like 3D nanostructured Co_3_O_4_@MnO_2_/NGO composite electrodes for electrochemical supercapacitor for the first time. The prepared Co_3_O_4_@MnO_2_/NGO and aqueous 6 M KOH solution have been utilized as working electrode and electrolyte respectively to evaluate the capacitive performance of the cell. Furthermore, the physicochemical properties of the Co_3_O_4_@MnO_2_/NGO electrode have been determined through Raman, XRD, XPS, FE-SEM, EDX, BET and FE–TEM analyses. The salient features of the present electrode system are discussed herein.

## Results and Discussions

**Figure 1:**
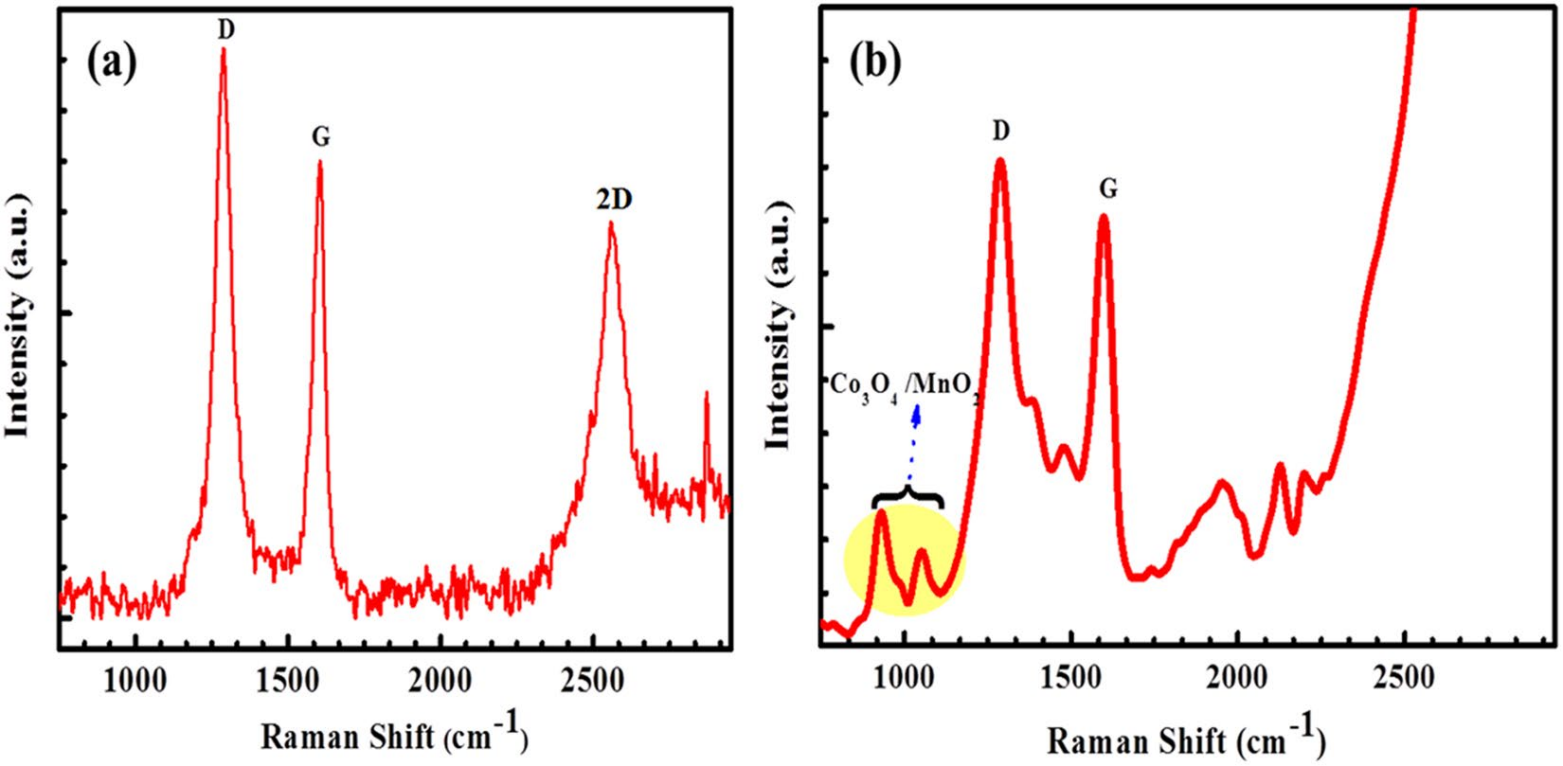
FT-Raman analysis of (**a**) NGO and (**b**) Co_3_O_4_@MnO_2_/NGO composite electrode.

The GO exhibits a considerable increase in the intensity ratio between D and G peaks have been evidenced through Raman analysis. In previous reports, the GO materials showed remarkable peaks at 1340 and 1590 cm^−1^, indicates that the D-band arises from the edge or defect sites of carbon and G band indicating the *sp*^2^ carbon of the graphene sheets^38^. The Raman spectra of the Co_3_O_4_@MnO_2_/NGO hybrid composite materials obtained *via* hydrothermal processing are shown in Fig. 1. The GO in the Co_3_O_4_@MnO_2_/NGO electrode structure is confirmed by the appearance of dominant peaks at 1345 cm^−1^ and 1589 cm^−1^ represents the D and G bands correspondingly. The D and G bands are meant for disordered *sp*^3^ carbon and well-ordered in-plane sp^2^ carbon bonds respectively which are in great resemblance with our recent reported result on MWCNT/GO/NiCo_2_O_4_ hybrid electrodes^38^. Further, the acquired results are in greatly concurrent with other previous reported literatures on graphene based composite materials^39,40^.

**Figure 2:**
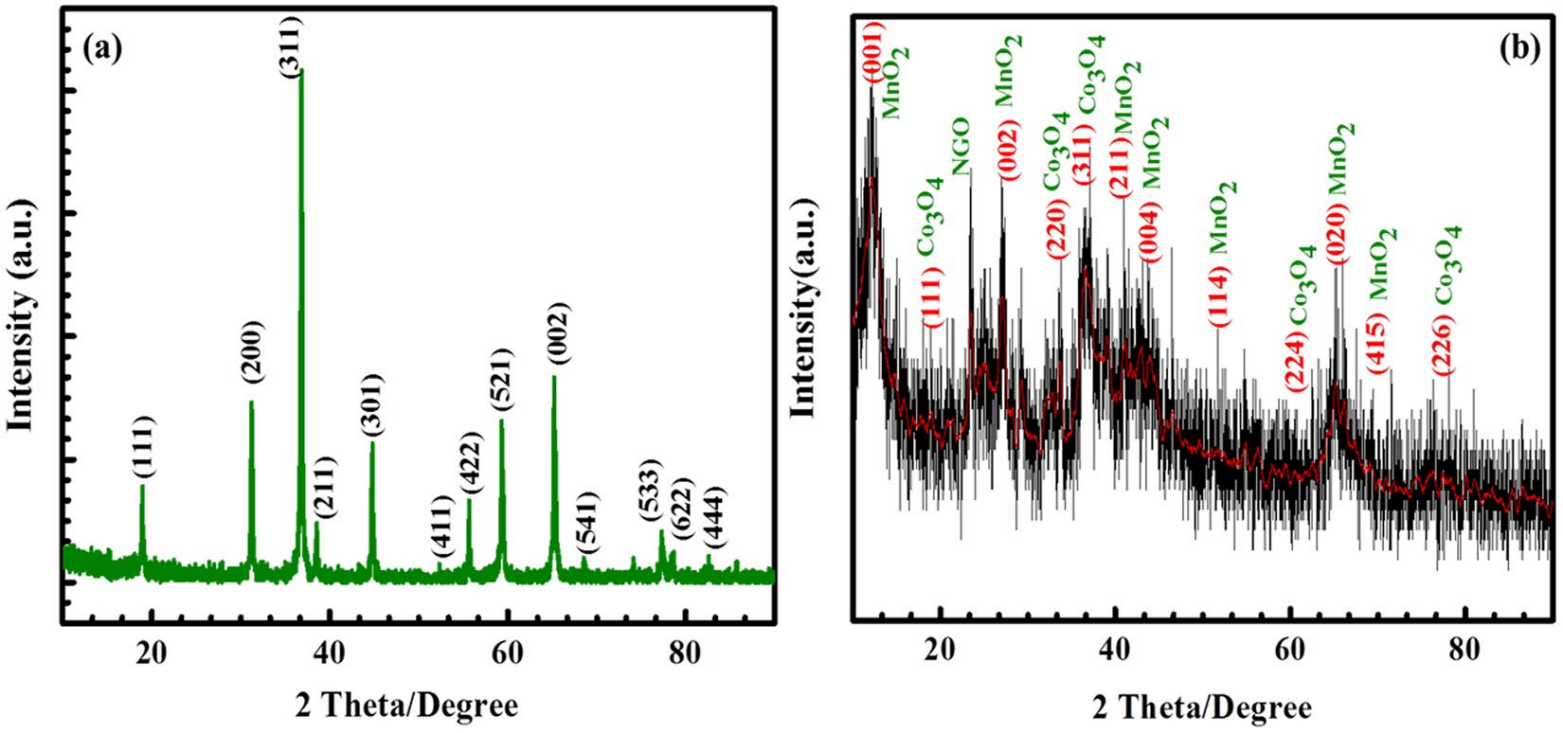
XRD results of (**a**) NGO/Co_3_O_4_ and (**b**) Co_3_O_4_@MnO_2_/NGO hybrid composites.

The XRD analysis of graphite, graphene oxide, reduced and nitrogen graphene oxides were previously reported in the literature^40^. The graphite powder showed characteristic diffraction peak at 2θ = 26.50°, and the corresponding layer-to-layer distance was ~0.36 nm. After the oxidation of graphite powder to GO in the presence of KMnO_4_, the diffraction peak shifted towards left at angle of 2θ = 10.40°, and the layer distance of ~0.89 nm confirmed the GO structure. In addition, the diffraction peak shown at around 2θ = 43° represents the graphene oxide with a turbostratic disorder behaviours^41–43^. In the present study, the results for the Co_3_O_4_@MnO_2_/NGO hybrid composite are shown in Fig. 2(a,b). It is observed from the Fig. 2(a,b) that the XRD patterns of Co_3_O_4_/NGO and Co_3_O_4_@MnO_2_/NGO hybrid composites consists of clearly distinguished peaks and the corresponding planes exactly matches well with the JCPDS PDF file data [42–1467]. The peaks at 18.96°, 31.20°, 36.74°, 38.28°, 44.72°, 55.44°, 59.20°, 65.10°, 77.12°, and 82.27° corresponds to (111), (22 0), (311), (222), (4 00), (422), (511), (44 0), (533), and (622) reflection planes of Co_3_O_4_/GO and pure Co_3_O_4_. The pattern resembles with the spinel Co_3_O_4_ structure and matches well with the Joint Committee on Powder Diffraction Standards (JCPDS) card [42–1467]. In addition, the analysis confirms that the crystal structure of the Co_3_O_4_ was maintained during the formation of the MnO_2_ on the Co_3_O_4_ nanoparticles in the core shell-like structure. The MnO_2_ nanoparticles are arranged in birnessite δ-MnO_2_ with broad and weak peaks at 12.52° (001), 25.26° (002), 36.20° (111), and 65.64° (020) due to the formation of Co_3_O_4_@MnO_2_ core-shell nanoparticles in the hybrid composite with good agreement of the birnessite-MnO_2_ (JCPDS No. 80–1098). The broad graphitic (002) and weak (100) peaks are proven as GO materials, and the disorderedly stacked GO sheets in the hybrid composite^44,45^.

**Figure 3:**
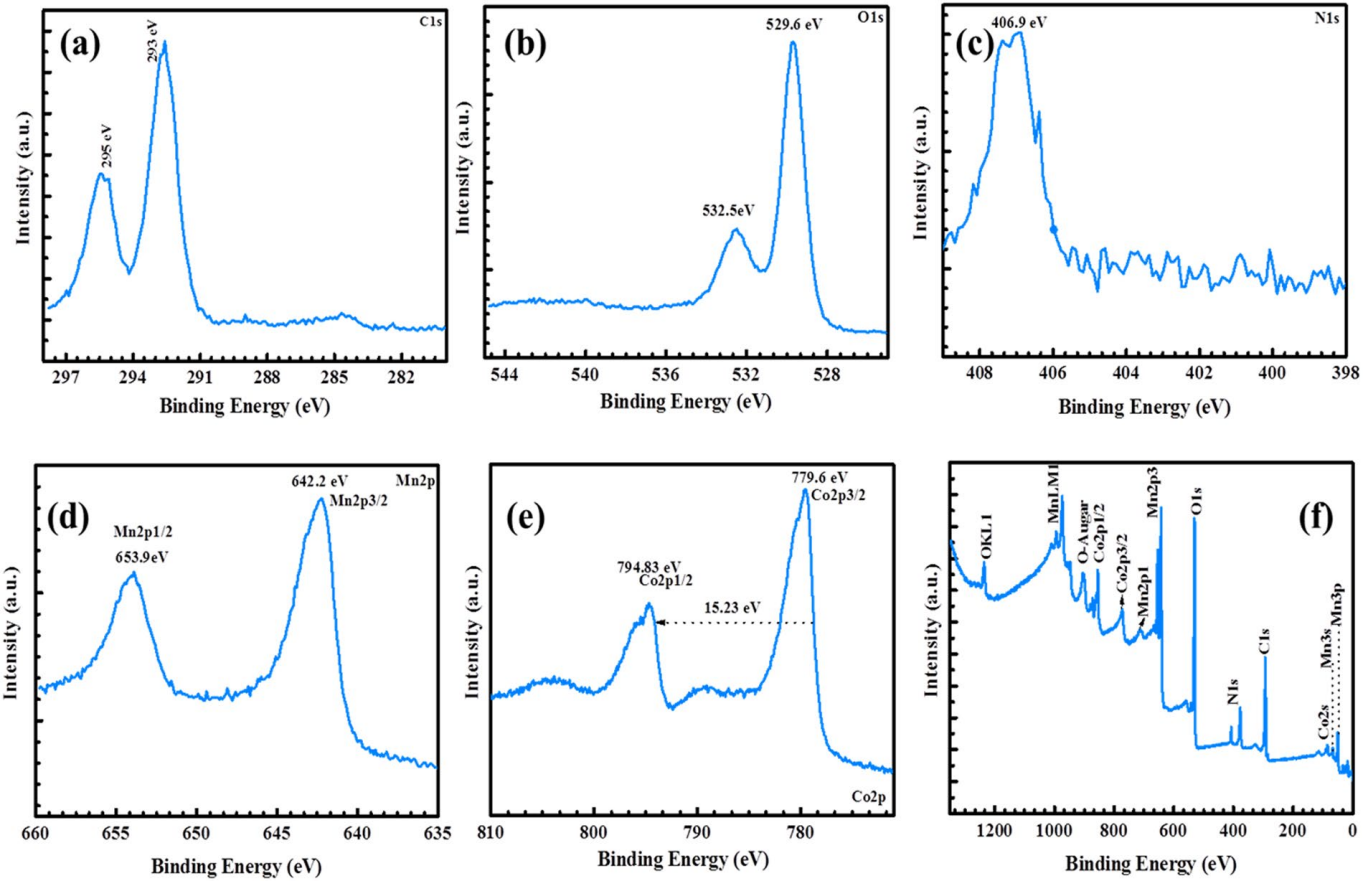
XPS results (**a**) C1s (b) O1s (**c**) N1s (**d**) Mn 2p (**e**) Co2p energy levels of hybrid composite.

The elements and chemical states of the Co_3_O_4_@MnO_2_/NGO composite have been studied through XPS analysis and the resultant results are depicted in Fig. 3. As shown in Fig. 3, the Mn 2p, Co 2p, C 1 s, O 1 s and N 1 s are found with their corresponding binding energies (BE). In addition, the XPS spectrum of C 1 s peaks corresponds to C 1 s (284–295 eV), O 1 s (529.6 eV), O* 1 s (532 eV) from Mn and Co oxides, N 1 s (398–406.9 eV) present in the hybrid composite. Therefore, the nitrogen (N 1 s) spectrum can usually be deconvoluted into three individual peaks, namely pyridine nitrogen (N), pyrrolic nitrogen (N) and graphite nitrogen (N), as confirmed in the hybrid composite. Besides the O 1 s peak and Co 2 s peak, two distinct peaks located at binding energies of 642.0 and 653.1 eV were observed in the Mn 2p core level spectrum, indicates that the Mn 2p_3/2_ and Mn 2p_1/2_ in manganese oxide present in the hybrid composite. The peak values are in concurrent with the earlier report of MnO_2_, indicating a +4 oxidation state of Mn^46^. These results provide direct evidence of Co_3_O_4_@MnO_2_ core shell nanoparticles in the hybrid composite obtained via thermal reduction process.

**Figure 4:**
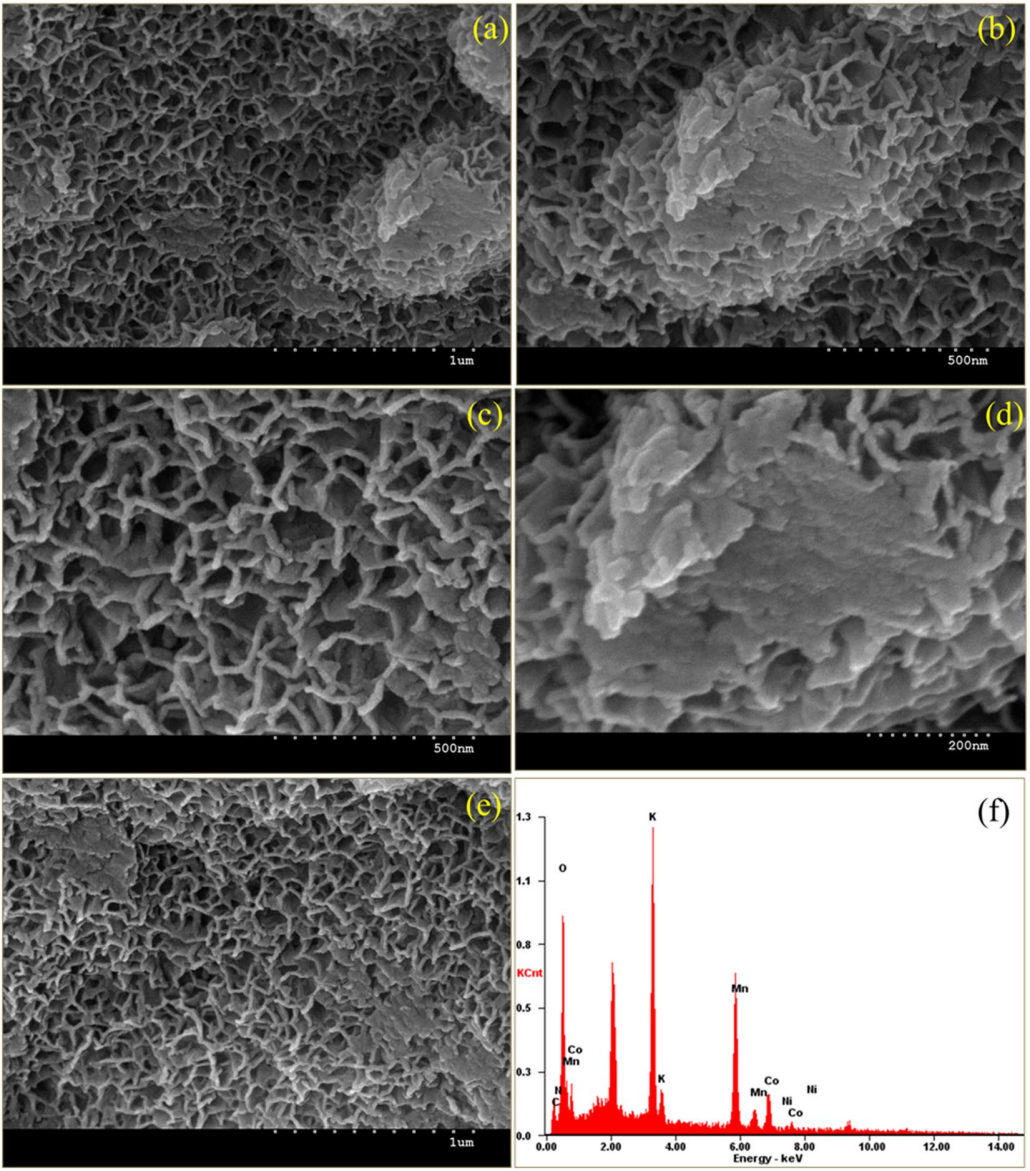
(**a**–**e**) FE-SEM morphology and (**f**) SEM-EDX of Co_3_O_4_@MnO_2_/NGO hybrid composites.

The surface morphology of the prepared Co_3_O_4_@MnO_2_/NGO electrode has evidenced through SEM analysis. The SEM images at different magnifications of Co_3_O_4_@MnO_2_/NGO are shown in Fig. 4(a–e). Its corresponding EDX spectrum is provided in Fig. 4(f). As it can be seen that the compact Co_3_O_4_@MnO_2_/NGO sheets grew vertically on the surface and assembly of these sheets in turn form a hierarchical porous structure as shown in Fig. 4(a–e). The obtained results strongly supports the results obtained from HR-TEM analysis which will be discussed further in the later section.

**Figure 5:**
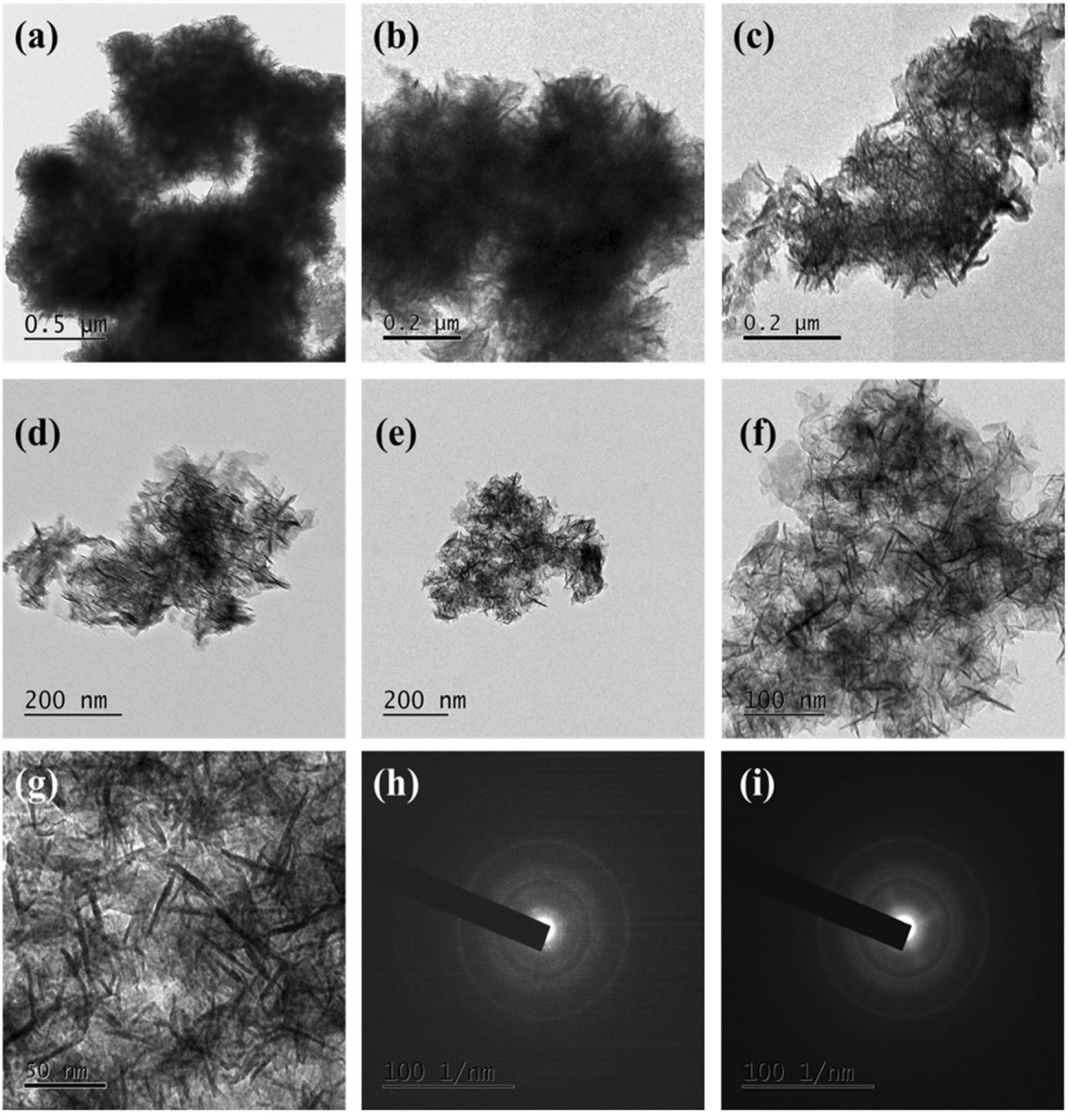
(**a**–**g**) FE-TEM images of Co_3_O_4_@MnO_2_/NGO at different particle sizes and (**h**,**i**) SAED array of Co_3_O_4_@MnO_2_/NGO.

Figure 5(a–g) shows the HR-TEM morphology and Fig. 5(h,i) the SAED patterns of Co_3_O_4_@MnO_2_/NGO hybrid nanocomposite.

The Co_3_O_4_@MnO_2_ electrode possess hierarchical flower-like morphology as represented Fig. 5 with the particle size in the range between 10 and 20 nm and decorated homogeneously over the NGO surface. The SAED patterns in Fig. 5(h,i) specify an ordered rings, which is owe to be the hierarchical flower like structure of Co_3_O_4_@MnO_2_.

**Figure 6:**
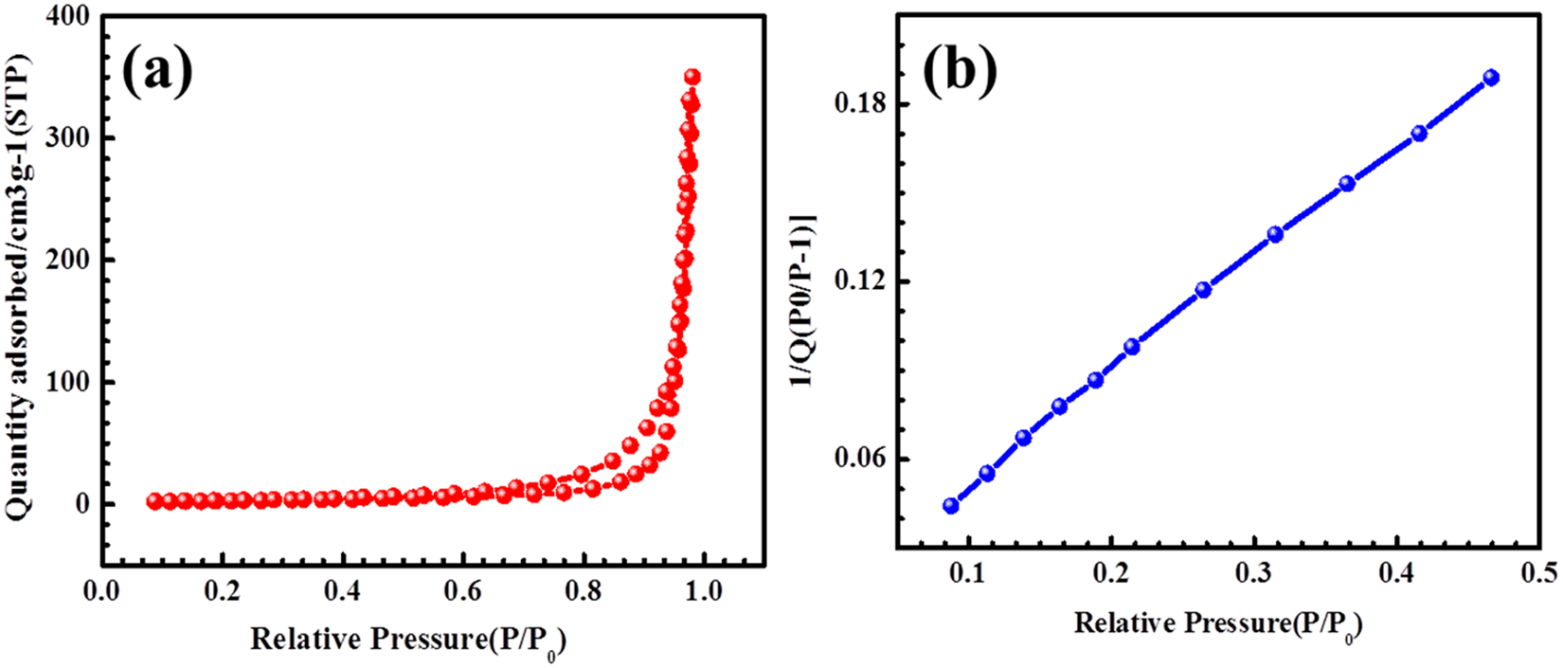
BET analysis of Co_3_O_4_@MnO_2_/NGO electrode.

Based on the pore structure, the active electrode surface of the porous carbon electrode for EDLCs is highly useful for supercapacitors in the presence of various electrolytes. Therefore, the porous structure of the porous carbon materials influences the energy/power densities in the electrochemical properties. Cobalt (Co) and manganese (Mn) electrodes are highly desirable for supercapacitor or batteries applications with enhanced surface properties^47^. The BET results of Co_3_O_4_@MnO_2_/NGO at 77 K are displayed in Fig. 6.

The N_2_ adsorption-desorption isotherm characteristics of the Co_3_O_4_@MnO_2_/NGO hybrid composite shows type II hysteresis loop as shown in Fig. 6(a). The surface area, pore volume, pore area and pore diameter values are ~350 m^2^/g, 0.55 cm^3^/g, 44 m^2^/g and 60 A°respectively. Furthermore, the surface area of Co_3_O_4_@MnO_2_/NGO increases, and the electrochemical behavior, such as the specific capacitance and cyclic stability, increases when compared to previously-reported hybrid composites^48–50^.

**Figure 7:**
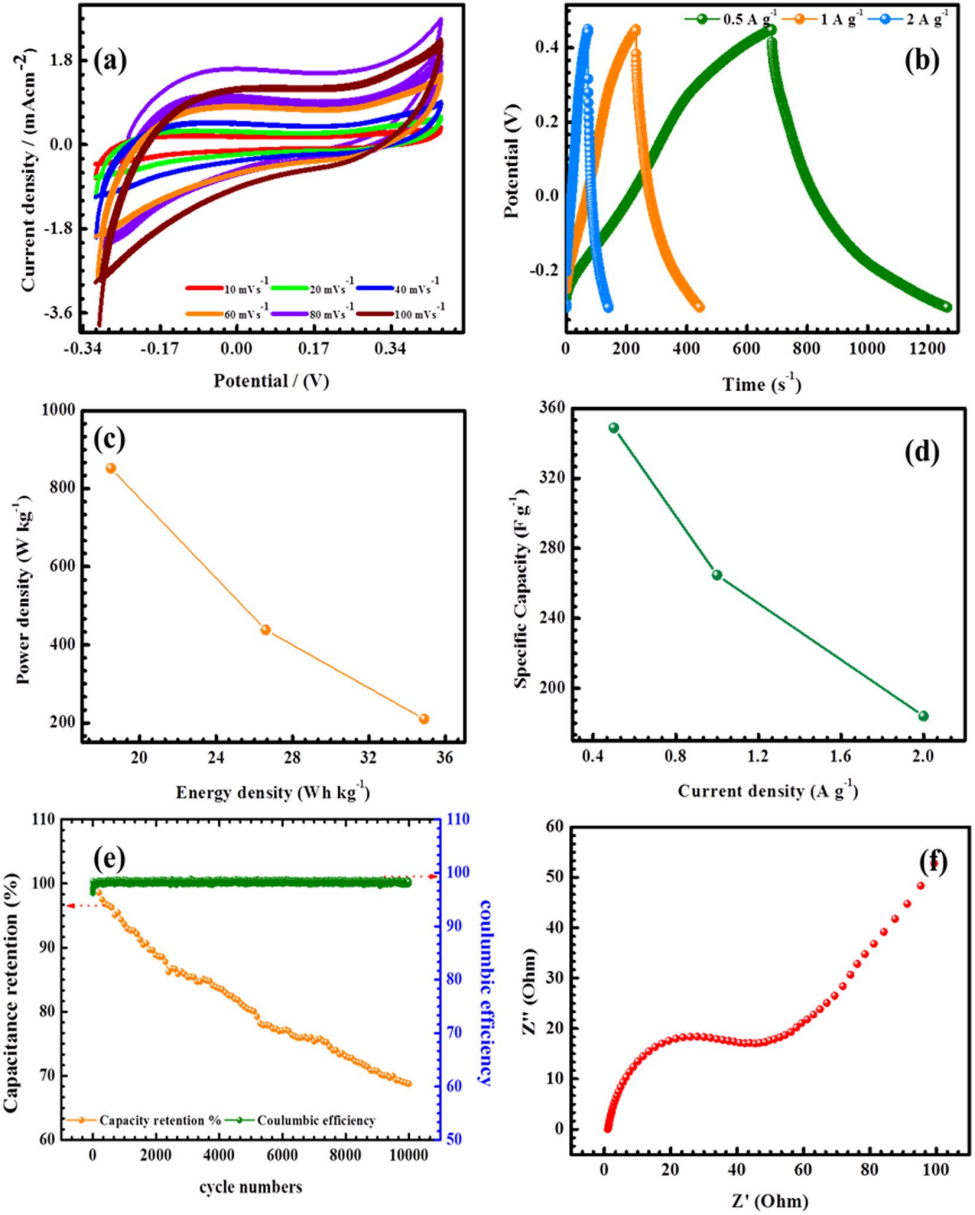
(**a**) CV plots of the Co_3_O_4_@MnO_2_/NGO at different scan rates in the range between 10 to100 mV s^−1^, (**b**) Galvanostatic charge-discharge behavior of the Co_3_O_4_@MnO_2_/NGO at various current densities, (**c**) Specific capacitance *vs* Current density plot, (**d**) Power density as function of energy density (**e**) Capacity retention plot for 10000 cycles (**f**) Nyquist impedance plot of the cell at ambient temperature.

The electrochemical properties of various nanostructured carbon-based Co_3_O_4_, MnO_2_, Co_3_O_4_@MnO_2_ core/shell and its electrochemical properties were previously reported in the literature^48–50^. This cobalt (Co_3_O_4_) and MnO_2_ materials clearly shows the redox behaviors of the metal oxide and different oxidation states (Co^3+/^Co^4+^) in the presence of a strong electrolyte *via* electrochemical reactions. Based on previous reports, the electrochemical properties of the Co_3_O_4_@MnO_2_/NGO hybrid composite have been investigated in a three-electrode configuration for CV, GCD and EIS experiments. The typical three-electrode cell containing Co_3_O_4_@MnO_2_/NGO ternary hybrid composite, Ag/AgCl, and Pt electrodes as working, reference, and counter electrodes, respectively, was dipped in 6 M aqueous KOH solution at room temperature. These results of the as-prepared Co_3_O_4_@MnO_2_/NGO electrodes in the different scans rates from (10 to 100 ms/V) are shown in Fig. 7. The CV curves are almost similar at all scan rates, indicating the reversible nature of the hybrid composite electrodes. The shape of the CV peaks represents the charge-discharge mechanism of the Co_3_O_4_@MnO_2_/NGO hybrid electrode via faradic reaction (oxidation and reduction reaction) of the metal ions together with 6 M KOH to improve the rate of reaction. Therefore, the CV area represents the total charge accumulating through the Faradaic and non-Faradic reaction. The faradaic contribution involves ion migrations with a surface-bound redox capacitance, whereas the non -Faradaic process is and effect of the double layer capacitance. The electrochemical properties of GO, RGO, NGO, Co_3_O_4_, MnO_2_ and Co_3_O_4_@MnO_2_ hybrid composites have been reported in the literature^51–53^.

Figure 7 represents the CV results of the Co_3_O_4_@MnO_2_/NGO composite electrode. The results of the CV curves for all electrodes shows a symmetrical behavior of the ideal capacitive properties with a quasi-rectangular shape due to the excellent capacitive nature of the electrodes. A close observation of the CV results of the hybrid composite electrodes indicates a more rectangular behaviour and a relative increment in the current level compared to that of pristine cobalt and manganese oxide materials^51–53^. These properties of the hybrid composites show the overall specific capacitance due to the combined contribution from EDLC and the pseudo-capacitance behaviour, and its related reversible reaction of Co_3_O_4_@MnO_2_ nanoparticles in the presence of a strong electrolyte. Based on the reversible reaction, the CV measurements have conducted at various scan rates, and the relative current response occurred with an ideal capacitive response of the Co_3_O_4_@MnO_2_/NGO composite.

It is perceived from the CV plot that the anodic and cathodic peaks are increases with increasing current density and sweep rates and the position of the peaks remains unaltered which indicates the ions are migrated in the both directions. Hence, the redox reactions are ensues at the electrode-electrolyte interfaces in presence of the strong electrolyte. These results show a rectangular behaviour with certain deviation due to the hydroxyl functional groups present in the NGO materials. Therefore, the CV results of the NGO materials are much larger then graphene-based materials^54,55^, which indicates the greater electrochemical properties of NGO. The specific capacitance values are calculated from Eq. (1) and these values are compared with earlier reports which are due to the surface properties of the electrolyte solution which reduces the internal resistance *R*_*i*_ thereby increase the pseudo-capacitance nature of the electrode material^54,55^. The CV results of (Fig. 7(a)) indicate that various scan rate of (10, 20, 40, 60, 80 and 100) mV s^−1^, around 95% of the initial capacitance has retained. These enhanced performances of the hybrid composite represent the following features in the electrochemical reaction. The first one is, the Co_3_O_4_@MnO_2_/NGO containing the more electroactive sites and exist as both double layer and pseudo-capacitance nature whereas the second one is due to decrement in internal resistance of the electroactive materials for enhancement of electrical conductivity^50–55^.

Furthermore, the results of the galvanostatic charge-discharge (GCD) profile explained the practical applications of Co_3_O_4_@MnO_2_/NGO hybrid composite. Figure 7(b–e) represents the GCD curves of the Co_3_O_4_@MnO_2_/NGO electrode using 6 M KOH solution at different current densities under ambient temperature. The GCD from Fig. 7(b) demonstrates that it consists of triangular shape-voltage profile which confirms the existence of the EDLC and pseudo-capacitance nature and is in concurrent with the results obtained from CV analysis. The Co_3_O_4_@MnO_2_/NGO electrodes offer the maximum discharge time than that of the pristine cobalt and manganese composites^30–37,56^, which indicate that the capacity has been stored for a prolong time by the prepared Co_3_O_4_@MnO_2_/NGO electrodes. The specific capacitance is calculated from Eq. (2) and it shows values of 347 Fg^−1^, 264, and 184 F g^−1^ at 0.5, 1, and 2 A g^−1^ respectively. The specific capacitance values of Co_3_O_4_@MnO_2_/NGO are quite high as compared to pristine Co_3_O_4_ and MnO_2_ composites which are because of its higher electrical conductivity, migration of ions in electrolyte solution, and electrical double layer charge storage capacity. The specific capacitance decreases with increase in current density in the order of (0.5, 1, and 2) A g^−1^. In addition, the electrochemical capacitance values are given in the (Table 1) with those of previously reported Co_3_O_4_@MnO_2_/NGO hybrid composites prepared from cobalt and manganese materials^46–55,57^.

**Table 1:** Comparison of the supercapacitor values of various nanostructured of cobalt oxides (Co_3_O_4_), Manganese oxides (MnO_2_) and Co_3_O_4_@MnO_2_/NGO electrodes reported in the literature.

Electrode material	Preparation method	Capacitance (F g^−1^)	Cyclic stability	Ref
Porous Cobalt oxide nanocomposite	Hydrothermal process	226.3 F g^−1^ at 10 mVs^−1^	24% loss after 5000 cycles	^46^
rGO/Cobalt oxide	Hydrothermal process	278.5 F g^−1^ at 200 m A g^−1^	9.4% loss after 2000 cycles	^47^
Cauliflower like Co_3_O_4_	Hydrothermal process	863 F g^-1^ at 1 mVs^−1^	No decay after 1000 cycles	^48^
Co_3_O_4_ decorated graphene	One –spot Solvothermal process	346 Fg^−1^ at 1 A g^−1^	15% loss after 50 cycles	^49^
Co_3_O_4_@graphene	Hydrothermal synthesis	415 Fg^−1^ at 3 A g^−1^	26% loss after 300 cycles	^50^
MnO_2_/RGO composite	Electrochemical deposition	125.93 Fg^−1^ at 10 mV s^−1^	20% loss after 5000 cycles	^51^
MnO_2_ on graphene	Hydrothermal	280 Fg^−1^ at 1 A g^−1^	9% loss after 10,000 cycles	^52^
Co_3_O_4_ nanotubes	Chemical deposition	273 Fg^−1^ at 0.5 A g^−1^	22% loss after 500 cycles	^53^
Cobalt tungstate (CoWO_4_)	Chemical precipitation reaction	1127.6 Fg^−1^ at 1 A g^−1^	24.3% loss after 3,000 cycles	^54^
Co_3_O_4_@MnO_2_ core shell nanostructure	hydrothermal approach	560 F g^−1^ at a current density of 0.2 A g^−1^	5% loss after 5000cycles	^55^
Co_3_O_4_@pt@MnO_2_	Nanowire arrays on the Ti substrate coating	497 F g^−1^ at 10 mV/s	No loss after 5000 cycles	^57^
**Co**_**3**_ **O**_**4**_ **@MnO**_**2**_ **/NGO**	**Thermal reduction process**	**347 F g** ^− **1**^ **at 0.5 A g** ^− **1**^	**31% loss after 10,000 cycles**	**This work**

Figure 7(c,d) illustrates the relation between power density and energy density (P vs E plot) and the capacity retention curve of the Co_3_O_4_@MnO_2_/NGO composite. The energy and power densities have been calculated from Eq. (3) and (4), and result is demonstrated in Fig. 7 (c,d) which indicates the long term cyclic stability of the active electrode materials for supercapacitor applications. Further, the cyclic stability of the Co_3_O_4_@MnO_2_/NGO hybrid composite electrodes have been confirmed by repeating the GCD analysis at 0.5 A g^−1^ for 10,000 cycles and its corresponding plot and values are provided in Fig. 7(e) and Table 1 respectively. After 10,000 cycles, the Co_3_O_4_@MnO_2_/NGO reached 69% of its initial specific capacitance, which indicates that the electroactive material possess excellent cycling stability and reversibility^46–55,57^. Table 1 compares the electrochemical properties of the Co_3_O_4_, MnO_2_ and Co_3_O_4_@MnO_2_ electrodes with the Co_3_O_4_@MnO_2_/NGO hybrid composite.

In order to evaluate the internal resistance and capacitance of the prepared Co_3_O_4_@MnO_2_/NGO electrode, EIS have been performed in the frequency range between 0.1 Hz and 100 KHz. The results have analyzed by using Nyquist plots, which denotes the frequency reaction of the electrode/electrolyte. Figure 7(f) indicates the EIS results of Co_3_O_4_@MnO_2_/NGO electrode and its corresponding equivalent series resistance (ESR) has been evaluated from the intercept of the X-axis at high frequency which is the total combination of the ionic resistance of electrolyte, intrinsic resistance of composite electrode, and contact resistance at current collector interface. The presence of straight line at lower frequency region and low ESR values may be due to the presence of Co_3_O_4_@MnO_2_ in Co_3_O_4_@MnO_2_/NGO electrode which enhanced the electrochemical properties of NGO. The value of ESR is ~40 Ω which indicates it has very small inherent resistance and the Warburg angle is higher than 45° at low frequencies which in turn due to the diffusion process at the electrode-electrolyte interfaces. Therefore, the charge transfer resistances of the electro active materials shows in lower frequency region were calculated 38–40 Ω respectively. The observed result strongly demonstrates the stable electrochemical properties of the Co_3_O_4_@MnO_2_/NGO electrodes.

## Conclusions

The controlled synthesis of hierarchal flower-like morphology of Co_3_O_4_@MnO_2_/NGO hybrid composites have been synthesized *via* thermal reduction and investigated its electrochemical behavior for high-performance supercapacitors. The Co_3_O_4_@MnO_2_/NGO composite electrode achieved the highest capacitance of 347 F g^−1^ at 0.5 A g^−1^. The excellent electrochemical behavior of the Co_3_O_4_@MnO_2_/NGO electrode was attributed to the high surface area and improved surface morphology, which facilitates the electron diffusion at the electrode/electrolyte interface. The long term cycling stability of the hybrid composite electrode was analyzed by subjecting the cell to GCD analysis at ambient conditions and it retained 69% of its capacitance after 10,000 charge–discharge cycles thereby demonstrating the excellent cyclic stability and reversibility of the prepared electrode material. The ESR and R_ct_ values were lower for the Co_3_O_4_@MnO_2_/NGO composite and hence it deeds as a promising candidate for high-performance supercapacitors.

## Materials and Methods

The graphite powder was gifted from Alfa Aesar, South Korea and used as such. Sulfuric acid (H_2_SO_4_,), N-methyl-2-pyrrolidone (NMP), nitric Acid (HNO_3_), polytetrafluorethylene (PTFE), potassium hydroxide (KOH), sodium nitrate (NaNO_3_), hydrogen peroxide (H_2_O_2_), cobalt nitrate (Co(NO_3_)_2_.6H_2_O), potassium permanganate (KMnO_4_), and Manganese acetate (Mn(CH_3_COO)_2_.4H_2_O) were used as the starting materials for cobalt and manganese oxides respectively. All the chemicals and solutions used in the present investigation were purchased from Sigma-Aldrich (South Korea).

### Graphene oxide (GO) preparation process

The GO powder was made by modified Hummers procedure and reported elsewhere^58,59^. In brief, the stoichiometric amounts of graphite flakes (3 g) and H_2_SO_4_ (20 ml), NaNO_3_ (1.4 g), were taken in a reaction flask and chilled to 0 °C. Afterward, the KMnO_4_ (0.18 g) was included in the reaction mixture slowly under room temperature. The resultant solution was subjected to stirring at 35 °C for about 12 h and then 30% H_2_O_2_ was added into the reaction mixture followed by neutral pH. The resulting GO materials were washed with water and ethanol and subjected to vacuum at 40 °C for 12 h. Finally, the finely dried GO powder was collected and kept in a desiccator.

### Synthesis of nitrogen-doped graphene oxide (NGO)

1 g of GO was dispersed in 200 mL of H_2_O under ultra-sonication treatment with high power pole for 4 h. The GO suspension thus obtained was filtered through Millipore (50 mm in diameter and 0.45 mm in pore size) filter and evaporated further at 95 °C for duration of 12 h. The purified GO was dispersed in water and ammonia (10 ml)/urea (1 g) and it was continuously stirred for 12 h at 90 °C. The resulted solution was dried at 180 °C and then purified several times with water-ethanol mixture. Finally, the NGO powder was subjected to calcination at 200 °C and it was collected for further characterization.

### Synthesis of Co_3_O_4_@MnO_2_/NGO hybrid composite

In a typical experiment, 0.3 g of NGO in 200 ml of water was stirred for about 2 h at 90 °C. Then, the stoichiometric amounts of cobalt nitrate [Co(NO_3_).6H_2_O] (3 g), manganese acetate [Mn (CH_3_COO)_2_ 4H_2_O] (1.5 g) and potassium permanganate (KMnO_4_) (1.5 g), urea (NH_2_CONH_2_) (1.2 g), and 30% aqueous ammonia solution were added and stirred continuously at 90  °C for 12 h. Initially, the solution was turbid due to the formation of cobalt /manganese hydroxides which was dissolved by adding excess ammonia/water. The transparent solution was then evaporated overnight at 90 °C under *vacuum*. The resulting product was purified using ethanol/water and calcined at 650 °C for 8 h *via* thermal reduction process.

### Materials Characterization

The NGO and Co_3_O_4_@MnO_2_/NGO composites were characterized by RM200 confocal Raman spectro microscopy scanned in the range of (100 to 400) cm^−1^ in presence of He and Ni laser beam. The XRD pattern of hybrid composite was studied by Rigaku Rotaflex (RU-200B) X-ray diffractometry, with CuK_α_ radiation (λ = 1.514 A°). The surface properties of Co_3_O_4_@MnO_2_/NGO were analyzed through field emission scanning electron microscopy (FE-SEM, Hitachi S-4800, and Japan) and filed emission transmission electron microscopy (FE-TEM, JEM-2010F). The elemental analysis of Co_3_O_4_@MnO_2_/NGO was examined using X-ray photoelectron spectroscopy (XPS, ESCALAB 250Xi, Thermo Fisher Scientific, and USA). The electrochemical properties of the hybrid composite had evaluated by Bio-Logics Science Instruments SAS Ltd (France). The CV experiment was conducted at ambient temperature prepared Co_3_O_4_@MnO_2_/NGO as a working electrode, the reference electrode was Ag/AgCl, and platinum wire was employed as the counter electrode. 6 M KOH served as electrolyte solution. The optimized potential window was in the range between −0.34 and 0.51 V and performed the tests at various scan rates (5 to 100) mV/s and current densities (0.5, 1, and 2) A g^−1^. The GCD was performed at current densities from (0.5, 1, and 2) A g^−1^. The electrochemical impedance spectroscopy (EIS) was measured in the frequency range between 0.1 Hz and 100 KHz with a signal amplitude of 500 mV.

### Preparation of working electrode (WE)

The WE was prepared by using stoichiometric amounts of Co_3_O_4_@MnO_2_/NGO, conductive carbon black, and PVDF in the ratio of 80:15:5 and formed as a slurry using NMP solvent. The slurry thus obtained was weighed (3 mg cm^−2^) and then coated over the stainless-steel substrate (1 × 1 cm^2^ active area). Finally the coated substrate was dried under vacuum at 110 °C for 12 h.

The specific capacitance of Co_3_O_4_@MnO_2_/NGO electrode was determined from Eq. (1)^60–62^.

1 $${\rm{C}}=\int \frac{Idt}{m{\rm{\Delta }}V}$$

where *I* denote the current, m represents the mass of the active material, *dt* the time interval and ${\rm{\Delta }}V\,\,$represents the potential difference.

The energy density E (Wh kg^−1^) and power density P (kW kg^−1^) was evaluated by means of following expressions 2 & 3,

2 $${\rm{E}}=\frac{1}{2}\times {\rm{C}}({{\rm{\Delta }}{\rm{V}}}^{2})/3.6$$

3 $${\rm{P}}={\rm{E}}\times 3600/{\rm{\Delta }}{\rm{t}}$$

In the above equations, ${\rm{\Delta }}{\rm{V}}$ and ${\rm{\Delta }}{\rm{t}}$ represents the voltage window and time interval for discharge process respectively^60^.
